# TRPM5 mediates acidic extracellular pH signaling and TRPM5 inhibition reduces spontaneous metastasis in mouse B16-BL6 melanoma cells

**DOI:** 10.18632/oncotarget.20826

**Published:** 2017-09-11

**Authors:** Toyonobu Maeda, Atsuko Suzuki, Kaori Koga, Chihiro Miyamoto, Yojiro Maehata, Shigeyuki Ozawa, Ryu-Ichiro Hata, Yoji Nagashima, Kazuki Nabeshima, Kaoru Miyazaki, Yasumasa Kato

**Affiliations:** ^1^ Department of Oral Function and Molecular Biology, Ohu University School of Dentistry, Koriyama 963-8611, Japan; ^2^ Department of Pathology, Fukuoka University School of Medicine and Hospital, Fukuoka 814-0180, Japan; ^3^ Department of Oral Science, Kanagawa Dental University Graduate School of Dentistry, Yokosuka 238-8580, Japan; ^4^ Department of Dentomaxillofacial Diagnosis and Treatment, Kanagawa Dental University Graduate School of Dentistry, Yokosuka 238-8580, Japan; ^5^ Oral Health Science Research Center, Kanagawa Dental University Graduate School of Dentistry, Yokosuka 238-8580, Japan; ^6^ Department of Surgical Pathology, Tokyo Women’s Medical University Hospital, Tokyo 162-8666, Japan; ^7^ Molecular Pathology and Genetics Division, Kanagawa Cancer Center Research Institute, Yokohama 241-8515, Japan

**Keywords:** MMP-9, acidic extracellular pH, TRPM5, melanoma, metastasis

## Abstract

Extracellular acidity is a hallmark of solid tumors and is associated with metastasis in the tumor microenvironment. Acidic extracellular pH (pH_*e*_) has been found to increase intracellular Ca^2+^ and matrix metalloproteinase-9 (MMP-9) expression by activating NF-κB in the mouse B16 melanoma model. The present study assessed whether TRPM5, an intracellular Ca^2+^-dependent monovalent cation channel, is associated with acidic pH_*e*_ signaling and induction of MMP-9 expression in this mouse melanoma model. Treatment of B16 cells with *Trpm5* siRNA reduced acidic pH_*e*_-induced MMP-9 expression. Enforced expression of *Trpm5* increased the rate of acidic pH_*e*_-induced MMP-9 expression, as well as increasing experimental lung metastasis. This genetic manipulation did not alter the pH_*e*_ critical for MMP-9 induction but simply amplified the percentage of inducible MMP-9 at each pH_*e*_. Treatment of tumor bearing mice with triphenylphosphine oxide (TPPO), an inhibitor of TRPM5, significantly reduced spontaneous lung metastasis. *In silico* analysis of clinical samples showed that high *TRPM5* mRNA expression correlated with poor overall survival rate in patients with melanoma and gastric cancer but not in patients with cancers of the ovary, lung, breast, and rectum. These results showed that TRPM5 amplifies acidic pH_*e*_ signaling and may be a promising target for preventing metastasis of some types of tumor.

## INTRODUCTION

Extracellular acidity, resulting from an increase in lactate concentration, a process known as “aerobic glycolysis” or “Warburg effect”, is a hallmark of solid tumors [1]. We have found that acidic extracellular pH (pH_*e*_) induces the production of matrix metalloproteinase-9 (MMP-9), the activity of which correlated with the metastatic ability of mouse B16 melanoma variants [2, 3]. In the intracellular signaling pathway for acidic pH_*e*_-induced MMP-9 expression, phospholipase D1 (PLD1) - mitogen activated protein (MAP) kinases (extracellular signal-regulated kinase1/2 and p38) - nuclear factor-κB (NF-κB) pathway plays an important role [4]. Acidic sphingomyelinase is also associated with NF-κB activation [5]. Importantly, acidic pH_*e*_-induced PLD1 activation is due to both the up-regulation of Ca^2+^ influx through voltage-gated Ca^2+^ channels and the activation of RhoA [5, 6]. Moreover, in some models, acidic pH_*e*_ has been reported to alter cell shape, from epithelial to fibroblastic [2, 7–9].

Melastatin (melastatin 1/TRPM1) is the first molecule of the transient receptor potential melastatin (TRPM) family to be identified. Although the level of expression of TRPM1 is inversely correlated with metastasis of human melanoma [10]. A recent study, however, showed that TRPM1 did not predict overall survival in patients with clinical AJCC stages I and II melanoma [11]. TRPM belongs to a non-selective cation channel family with six transmembrane structures. Although eight genes have been identified, TRPM4 and TRPM5 are the only Ca^2+^-activated nonselective monovalent cation channels, which carry Na^+^, K^+^, and Cs^+^ ions, and may be important for membrane depolarization that triggers neuronal responses [12, 13]. TRPM5 has been reported to be associated with sweet and umami, but not sour, taste signaling in taste buds in a heat-dependent manner [14, 15]. Interestingly, TRPM5 activity is affected by acidic pH_*e*_, resulting in a rapid reversible block in current (IC_50_ pH = 6.2) and a slower irreversible inactivation of current [13].

Tumor invasion and metastasis are regulated by membrane potential through potassium and sodium channels and exchangers, so that experimental inhibition of these channels successfully reduces tumor metastasis. For example, inhibition of the potassium channels, KCNK9 [16], Kv10.1 [17], hEag1 [18], and HERG1 [19] inhibited tumor metastasis, as did inhibition of the sodium channels, Na(v1.4) [20] and Na(v)1.5 [21] and the sodium proton exchanger, NHE1 [22, 23].

Although intracellular Ca^2+^, which is apparently increased by acidic pH_*e*_ [5], indirectly activates PLD1 through protein kinase Cα (PKCα), a conventional PKC isotype dependent on Ca^2+^ and diacylglycerol [24], the PKC activator phorbol-12-myristate-13-acetate (TPA) could not mimic acidic pH_*e*_-induced MMP-9 expression by mouse B16 cells at neutral pH_*e*_ [2], suggesting that elevation of intracellular Ca^2+^ affects other targets. Our preliminary experiments using K^+^- and Na^+^-sensitive fluorescent dyes, such as the K^+^-binding dye benzofuran isophthalate (PBFI-AM) and the Na^+^-binding dye benzofuran isophthalate (SBFI-AM), showed that PBFI-AM, but not SBFI-AM, dose-dependently inhibited acidic pH_*e*_-induced MMP-9 production. These findings prompted a determination as to whether TRPM5, a transient Ca^2+^-activated cation channel, is involved in the acidic pH_*e*_ signaling that induces MMP-9 expression in melanoma.

## RESULTS

### *Trpm5* mRNA is highly expressed in the metastatic B16 melanoma variant BL6

**Figure 1:**
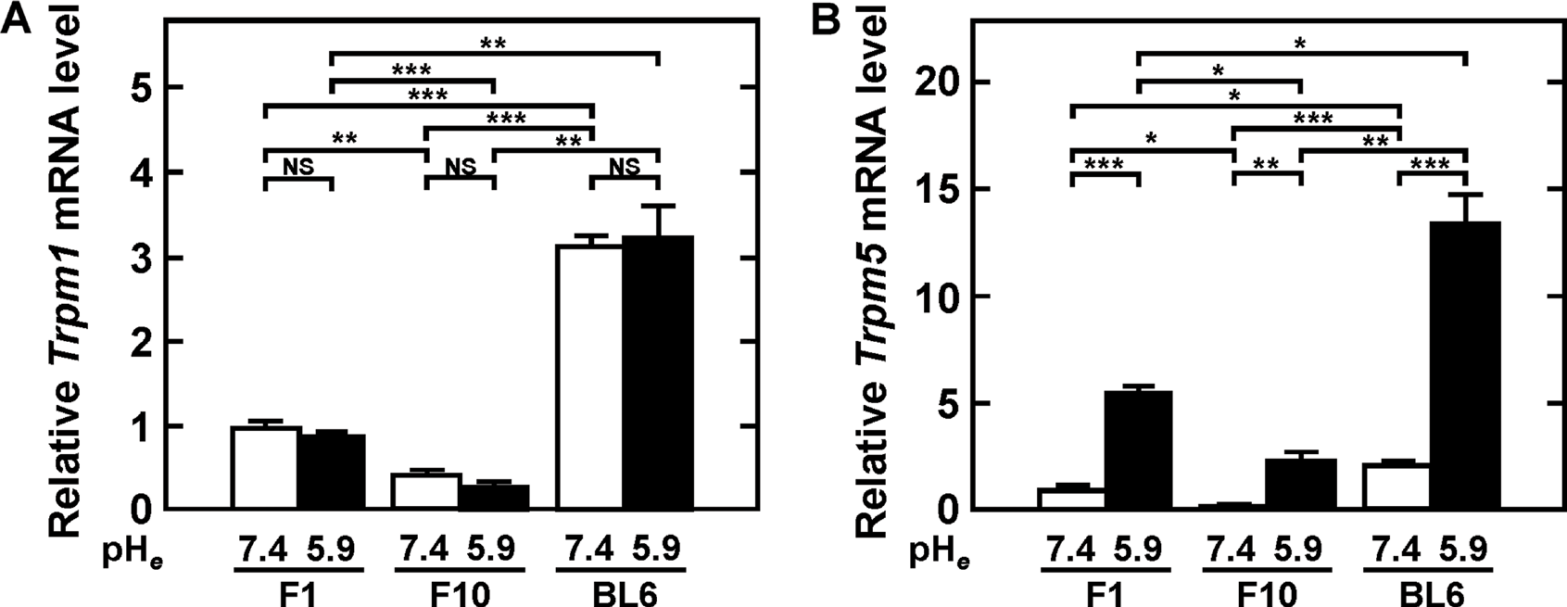
Expression of *Trpm1* (**A**) and *Trpm5* (**B**) mRNAs by confluent cultures incubated with serum-free medium at the indicated pH for 24 h. Total RNA was extracted and reverse transcribed for RT-qPCR. Data were expressed, relative to the level of F1 cells at pH_*e*_ 7.4, as mean ± SE in triplicate assay. *P<0.05, **P<0.01, ***P<0.001. NS, not significant.

Levels of *Trpm5* mRNAs were compared in F1 (low metastatic), F10 (highly metastatic experimentally but not spontaneously) and BL6 (highly metastatic in both models) [10, 25, 26]. In addition, levels of *Trpm1* mRNA, a prototype of TRPM, were compared, as these levels were found to inversely correlate with metastatic ability [10, 25, 26]. Levels of *Trpm1* mRNA expression among B16 melanoma variants were in the descending order BL6, F1, and F10, but were not affected by acidic pH_*e*_ (Figure 1A). In contrast, *Trpm5* mRNA expression was detected in all three variants, being relatively high in BL6 cells but low in F1 and F10 cells. In addition, *Trpm5* mRNA expression was significantly induced in all variants by acidic pH_*e*_. Especially, the induction in BL6 cells was extremely stimulated by acidic pH_*e*_ (Figure 1B). All variant cell lines tested were positive for mRNAs encoding other subtypes of *Trpm*, except for *Trpm4b*, *Trpm6*, and *Trpm8*. Although levels of *Trpm3a* and *Trpm4a* mRNAs were higher at acidic than at neutral pH_*e*_, these levels did not correlate with the metastatic ability of B16 melanoma variants (data not shown).

### TRPM5 reduction inhibits acidic pH_*e*_-induced MMP-9 expression

**Figure 2:**
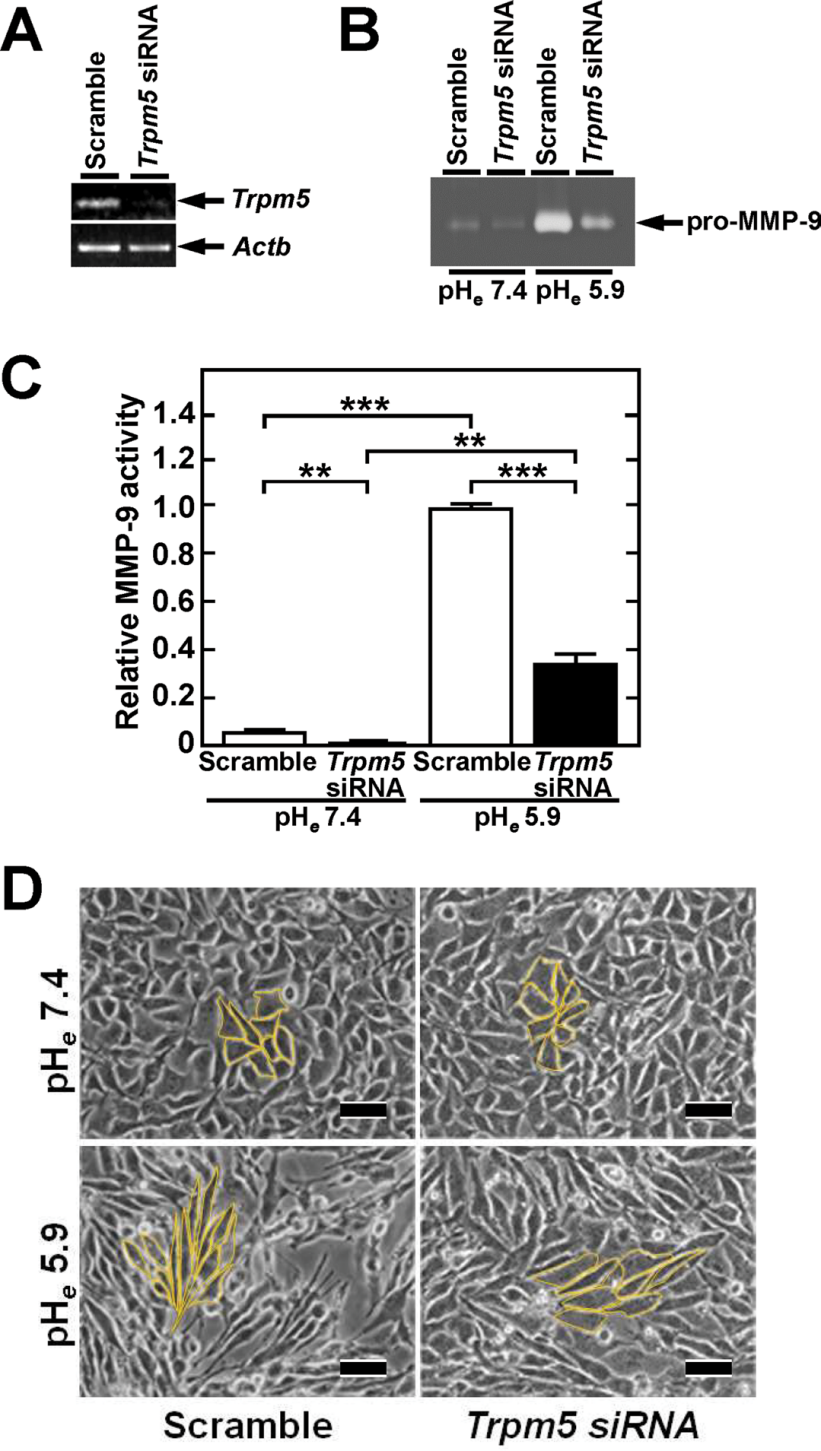
Introduction of *Trpm5* siRNA reduces acidic pH_*e*_-induced MMP-9 production and alters morphology to fibroblastic (**A**) Reduction of *Trpm5* mRNA by transfection of *Trpm5* siRNA. PCR product was separated on agarose gel and stained with ethidium bromide. Reduction rate by siRNA was quantified by qPCR (see Supplementary Figure 1). (**B**) Inhibition of acidic pH_*e*_-induced MMP-9 production by transfection of *Trpm5* siRNA. Following transfection, the cells were incubated in serum-free medium at neutral and acidic pH. The MMP-9 concentration of conditioned medium was analyzed by gelatin zymography. (**C**) Densitometric analysis of MMP-9 activity on zymogram in B (*n* = 3). Data were expressed relative to maximum induction at pH_*e*_ 5.9. (**D**) Acidic pH_*e*_ induced fibroblastic shape, but this change was inhibited by introduction of *Trpm5* siRNA. Representative results are shown. Bar, 50 µm. Cell shapes are highlighted in yellow line and their quantification is shown in Supplementary Figure 2.

To determine the association between TRPM5 and acidic pH_*e*_ signaling, we tested the effect of siRNA specific for *Trpm5* mRNA on MMP-9 production at acidic pH_*e*_. The siRNA for *Trpm5* markedly reduced pH_*e*_-induced MMP-9 expression (Figure 2A–2C and Supplementary Figure 1), suggesting that TRPM5 is involved in acidic pH_*e*_ signaling that induces MMP-9 production. In addition, *Trpm5* siRNA inhibited acidic pH_*e*_-induced cellular elongation, to a fibroblastic shape characteristic of changes associated with epithelial mesenchymal transition (EMT) (Figure 2D and Supplementary Figure 2).

### Enforced expression of TRPM5 amplifies acidic pH_*e*_ signaling

**Figure 3:**
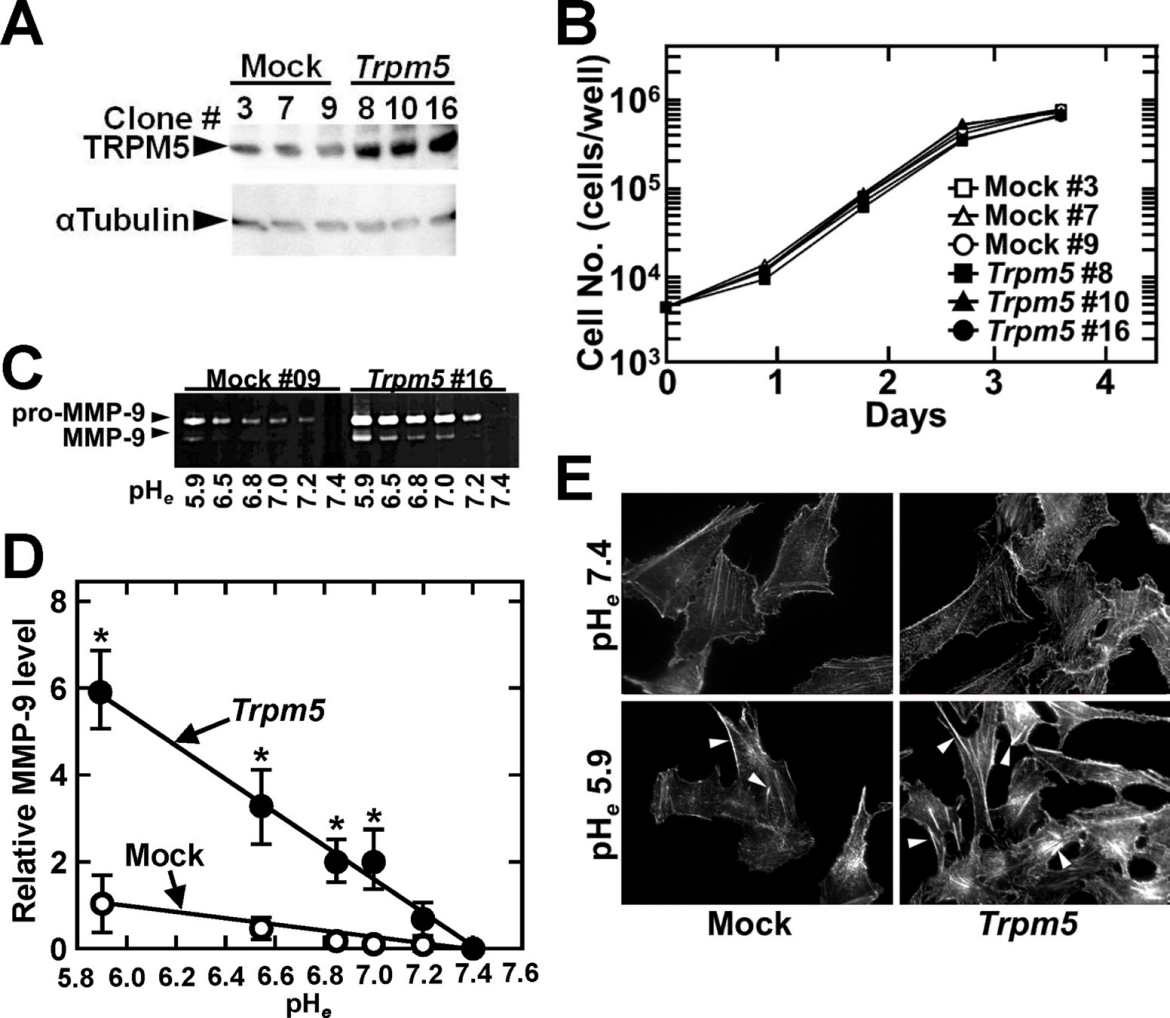
Constitutive expression of *Trpm5* mRNA does not affect cell growth but increases the induction of MMP-9 and actin reorganization by acidic pH_*e*_ (**A**) Western blotting of representative clones of *Trpm5*-expressing vector transfected cells. (**B**) Growth curves of the representative clones (*n* = 3). (**C**) Zymographic analysis of acidic pH_*e*_-induced MMP-9 secretion from clone #9 of mock and #16 of *Trpm5* transfectants. This panel shows application of 1/4 samples to see differences in expression. (**D**) Densitometric analysis of the results consisting of three-clone each transfectants: Mock (# 3, #7, and #9 in (C)) and *Trpm5* (#8, #10, and #16 in (C)). (**E**) Rhodamine-phalloidin staining. Arrowheads show actin stress fibers, whose quantification is shown in Supplementary Figure 3.

To determine whether TRPM5 can sensitize cells to acidic pH_*e*_, thereby inducing MMP-9 expression, a *Trpm5* gene expression vector was introduced into BL6 cells. Cell clones expressing high levels of TRPM5 protein were tested for the ability of acidic pH_*e*_ to induce MMP-9 (Figure 3A). As expected, cells expressing high TRPM5 levels showed an increase in acidic pH_*e*_-induced MMP-9 expression, but there was no effect on cell proliferation (Figure 3B–3D). Because the highest pH to induce MMP-9 production by both mock control and TRPM5-overexpressing cells was 7.2, these results suggested that the effect of pH on MMP-9 production was due to amplification of the acidic pH_*e*_ signal rather than to an increase in critical pH_*e*_. As reported previously [6], acidic pH_*e*_ increased stress fiber formation, an effect enhanced by the forced expression of the *Trpm5* gene (Figure 3E and Supplementary Figure 3). Because TRPM5 activity is thermo-dependent [14], we also tested the effect of incubation temperature on acidic pH_*e*_-induced MMP-9 production. The acidic pH_*e*_ induction of MMP-9 at 37°C was obviously reduced when cells were incubated at 30°C or 25°C while *Trpm5* and *MMP2* mRNA expression were not inhibited between neutral and acidic pH_*e*_ at each temperature, showing that channel activity of TRPM5 required to transduce acidic pH_*e*_ signaling (Supplementary Figure 4).

### Enforced expression of *Trpm5* induces experimental lung metastasis

**Figure 4:**
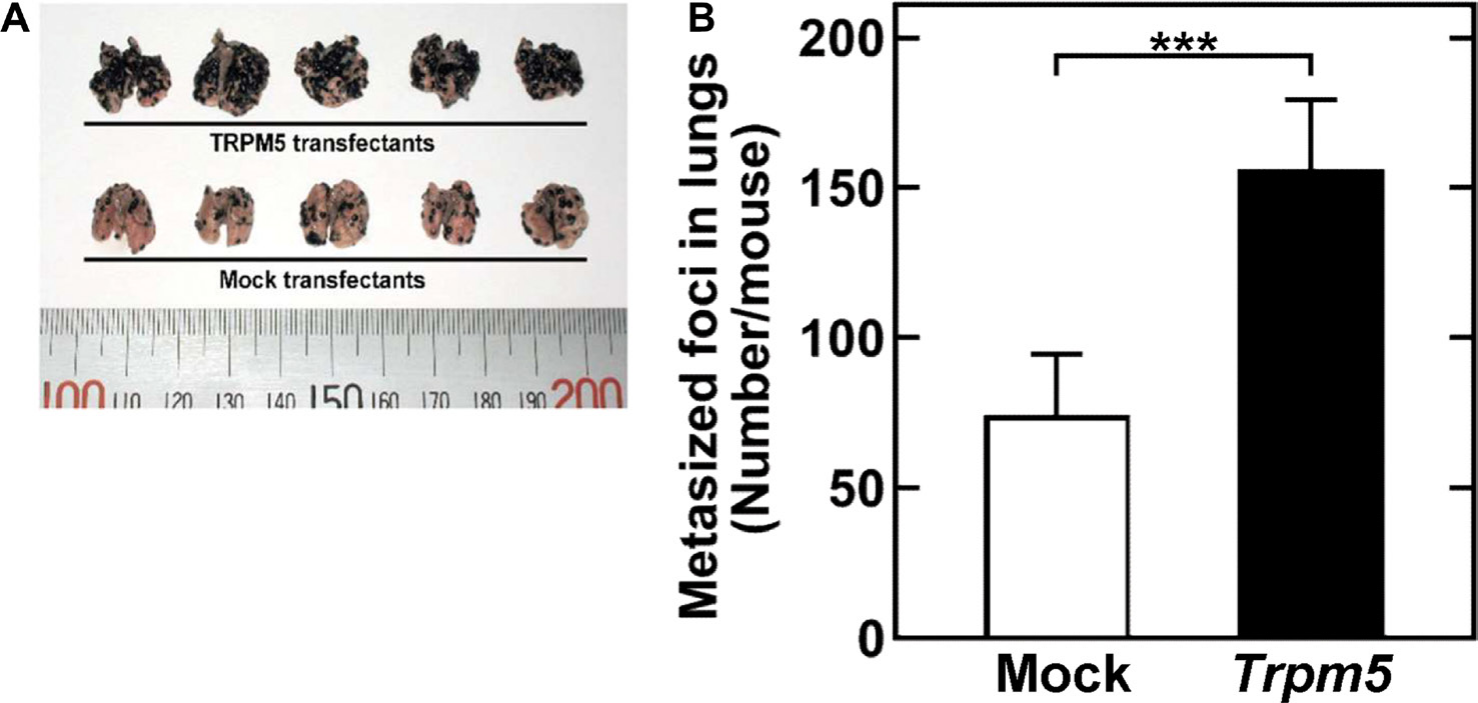
Constitutive *Trpm5* mRNA expression increases experimental pulmonary metastasis through tail vein injection of B16-BL6 cells Representative clones of cells transfected with empty vector (control) and *Trpm5*-expressing vector (*Trpm5*) were grown individually, pooled and injected into tail veins of mice. **(A)** Photograph of a lung 3 weeks after injection. **(B)** Metastasized foci at the lung surface. Data are represented as mean ± SE (*n* = 15). ***P < 0.01.

Injection of *Trpm5* transfectants into the tail veins of mice doubled the number of metastasized foci in the lungs observed following injection of mock transfectants (Figure 4). This result clearly indicated that TRPM5 plays an important role in the pulmonary metastasis of melanoma cells.

### Triphenylphosphine oxide (TPPO) treatment reduces acidic pH_*e*_-induced MMP-9 production and spontaneous metastasis of melanoma

**Figure 5:**
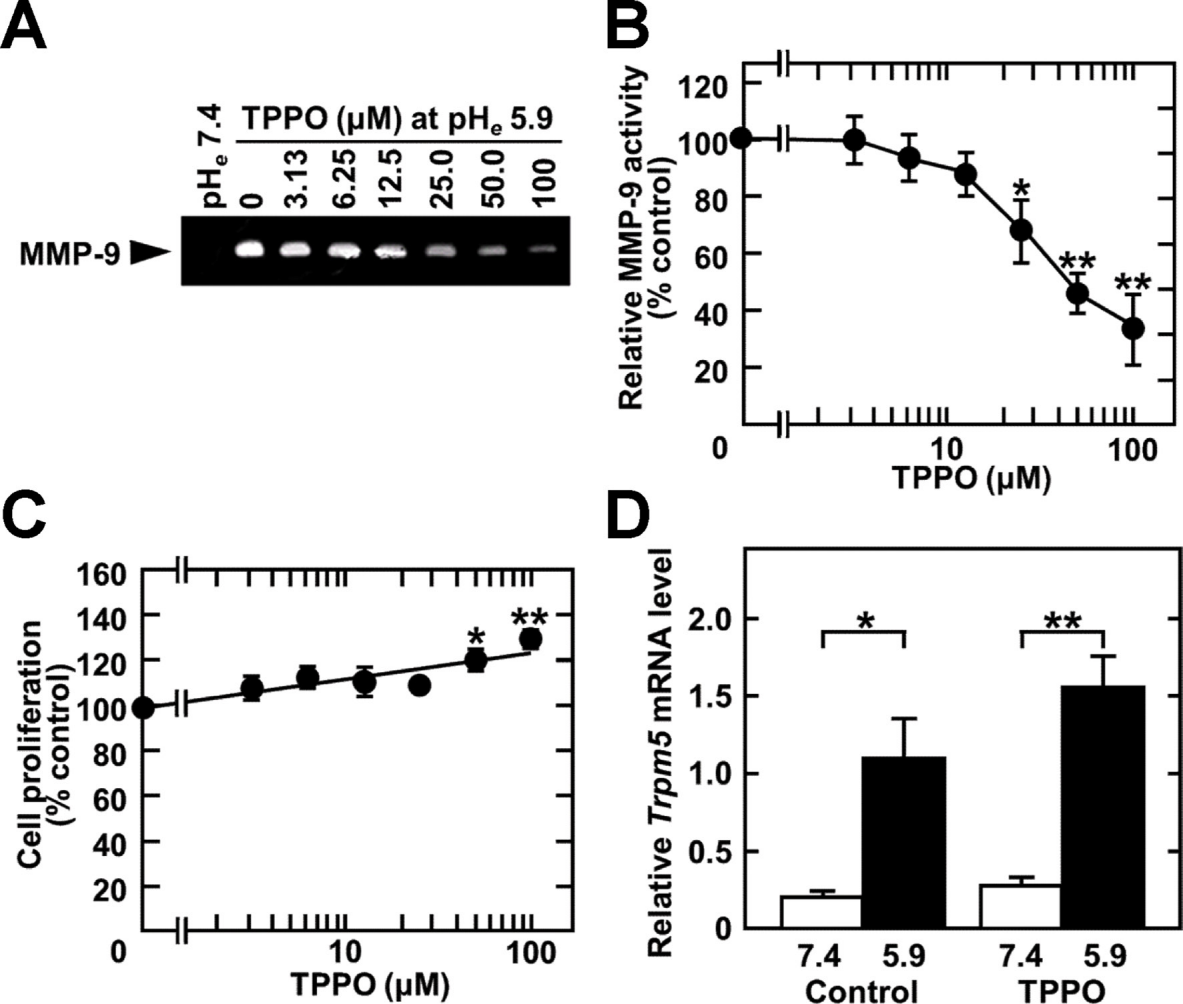
TPPO treatment inhibits acidic pH_*e*_-induced MMP-9 production Cells were treated with the indicated concentration of TPPO in serum-free medium for 24 h. (**A**) Conditioned media were concentrated and analyzed by zymography. (**B**) Densitometric analysis of the results in (A). (**C**) Cells were cultured with the indicated concentrations of TPPO in the presence of 10% FBS for 2 days, and cell survival was measured by the CCK8 assay. (**D**) Cells were pre-treated for 1 h and treated for a further 24 h with 50 mM of TPPO in the absence of serum. Total RNA was extracted, reverse-transcribed, and amplified by qPCR. Data are represented as mean ± SE (*n* = 3). *P < 0.05, **P < 0.01.

**Figure 6:**
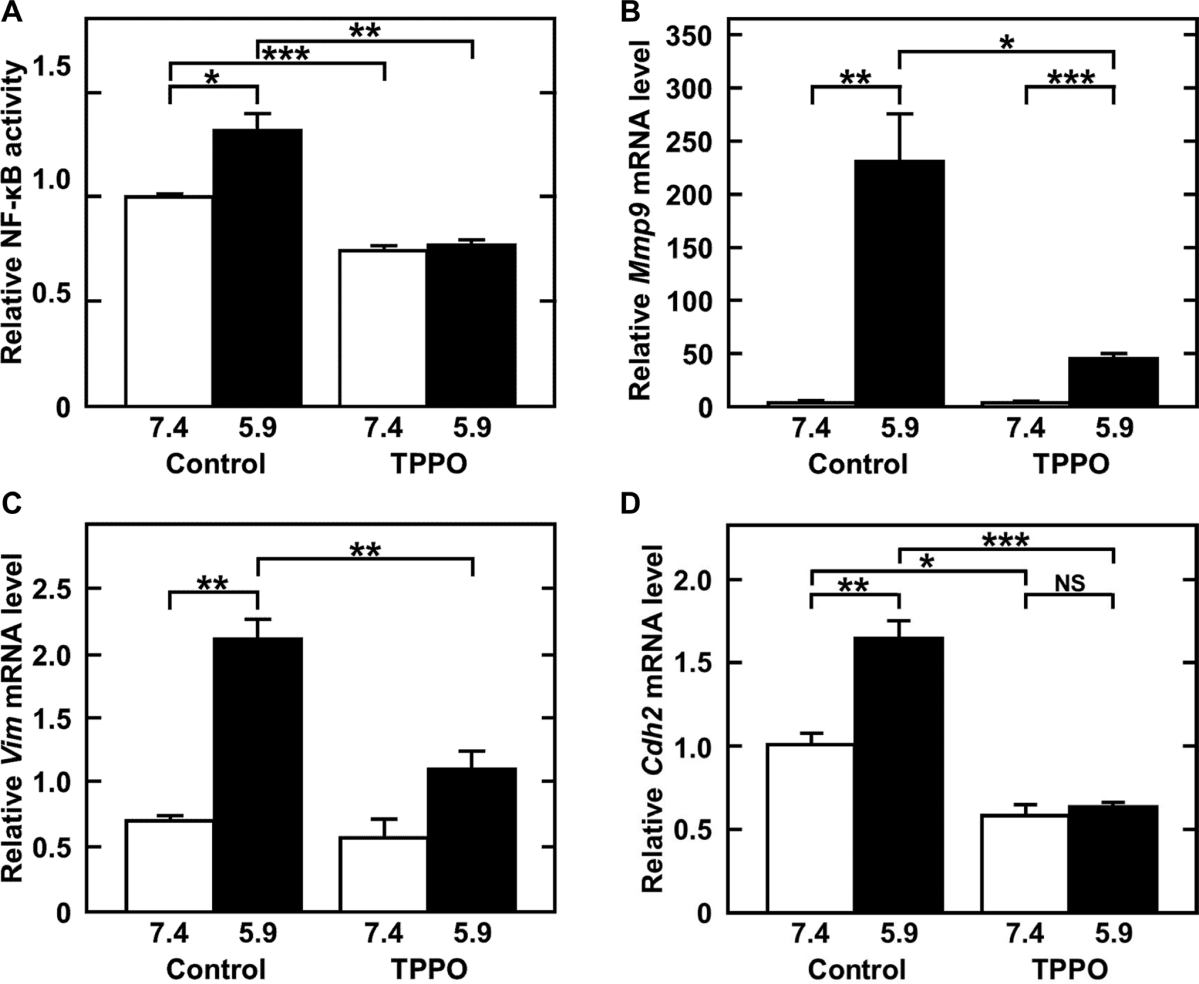
TPPO treatment inhibits acidic-pH_*e*_-induced NF-κB activity (**A**) and *Mmp9* (**B**), *Vim* (vimentin) (**C**), and *Cdh2* (N-cadherin) (**D**) mRNA levels. (A) Cells were transfected with NF-κB-driven luciferase vector, pre-treated with 50 mM of TPPO in serum-free medium at neutral pH_*e*_ for 1 h, and then treated with 50 mM of TPPO in serum-free medium at acidic pH_*e*_ for 24 h. Cell lysates were collected and NF-κB-driven luciferase activity measured. (B**–**D) Cells were pre-treated for 1 h and treated for a further 24 h with 50 mM of TPPO in the absence of serum. Total RNA was extracted, reverse-transcribed, and amplified by qPCR. Data are represented as mean ± SE (*n* = 3). *P < 0.05, **P < 0.01, ***P < 0.005.

**Figure 7:**
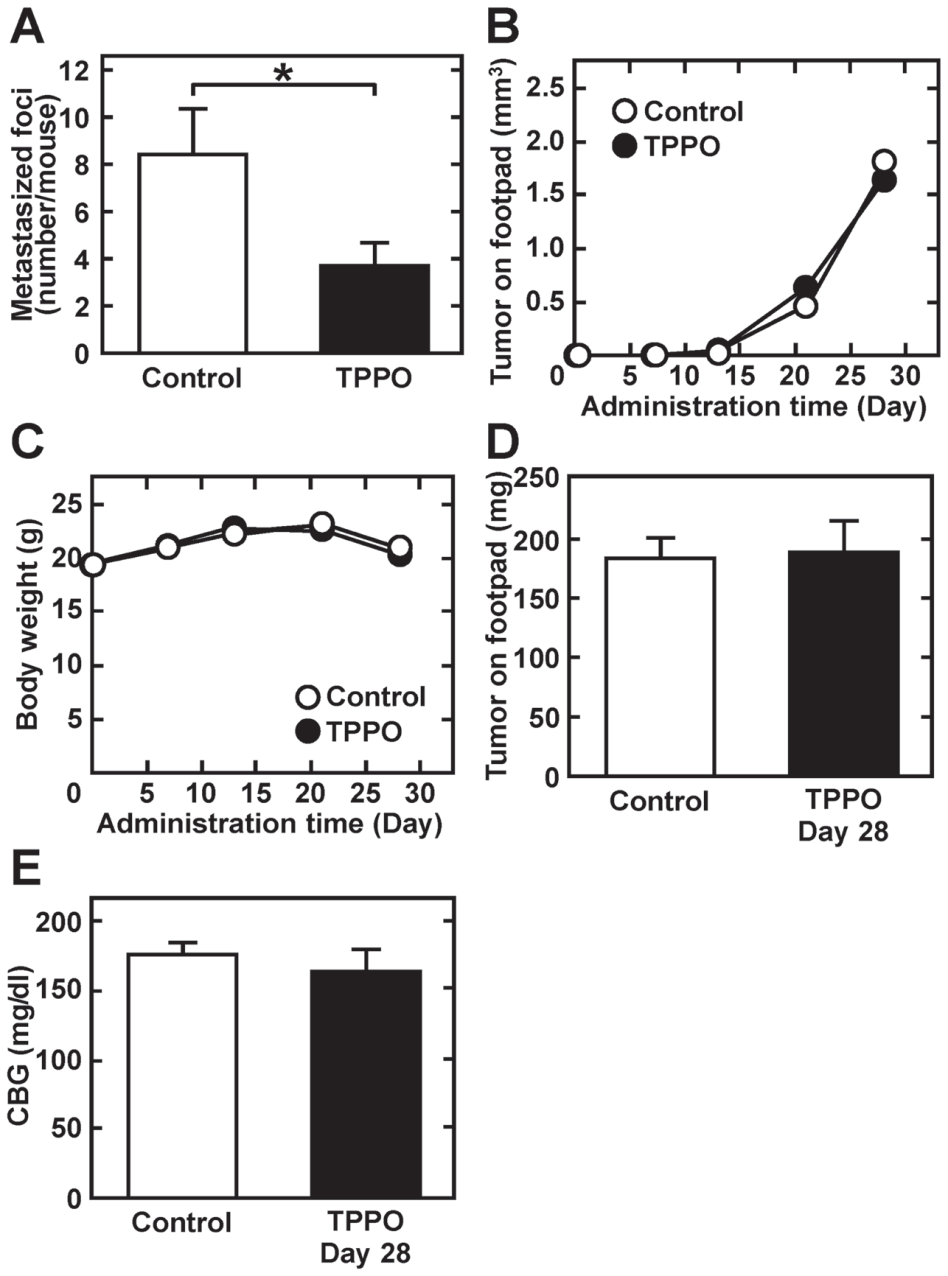
TPPO administration reduces pulmonary metastasis without affecting tumor growth, body weight, or CBG Cells were injected into the left footpads of mice. Beginning 2 days later, mice were subcutaneously injected with 10 mg/kg TPPO dissolved in DMSO every other day. Primary tumors were amputated 3 weeks later and treatment with TPPO continued for another 4 weeks. (**A**) Numbers of pulmonary metastasized foci (*n* = 17). (**B**) Volumes of primary tumors (*n* = 17). (**C**) Body weight (*n* = 17). (**D**) Tumor weight after the amputation of tumor tissue (*n* = 17). (**E**) CBG concentration (*n* = 3). Data are represented as mean ± SE. *P < 0.05.

The ability of chemotherapy targeting TRPM5 to prevent lung metastasis was assessed by evaluating the effect of TTPO, a pharmacologic inhibitor of TRPM5 activity [27], on acidic pH_*e*_-induced MMP-9 expression. As expected, TPPO dose-dependently inhibited acidic pH_*e*_-induced MMP-9 production (IC_50_: 41 mM after 24 h) (Figure 5A and 5B), but did not reduce cell viability. Rather, high concentrations of TPPO slightly promoted cell growth (Figure 5C). Although acidic pH_*e*_ induced *Trpm5* gene expression, TPPO did not reduce the level of *Trpm5* mRNA (Figure 5D). However, it inhibited acidic pH_*e*_-induced NF-κB activity and expression of EMT-related genes, such as *Mmp9*, *Vim* (vimentin), and *Cdh2* (N-cadherin) (Figure 6). These results suggested that EMT-related gene expression was up-regulated by acidic pH_*e*_ through TRPM5 and that pharmacological inhibition of TRPM5 activity was effective in preventing acidic pH_*e*_-induced EMT. Because acidic pH_*e*_ induced *Trpm5* mRNA expression and this induction was not inhibited by TPPO, the mechanism involved in the up-regulation of *Trpm5* mRNA expression differs from that involved in the induction of *Mmp9* and other genes. Finally, TRPM5 was targeted in melanoma bearing mice by treatment with TPPO. B16-BL6 cells were inoculated into the left footpads of these mice, followed by subcutaneous injection of TPPO every other day. Three weeks after cell injection, and while continuing TPPO administration, the limb containing the primary tumor was amputated. Four weeks later, the mice were sacrificed and the numbers of metastatic foci were counted. TPPO treatment clearly prevented pulmonary metastasis of B16-BL6 cells (Figure 7A), but had no effect on the primary tumor mass or on body weight (Figure 7B–7D). Because TRPM5 has been reported associated with insulin secretion [28], casual blood glucose (CBG) levels were measured after TPPO treatment. TPPO did not significantly affect blood glucose concentrations in these mice (Figure 7E).

### Expression of TRPM5 in tumor patients

**Figure 8:**
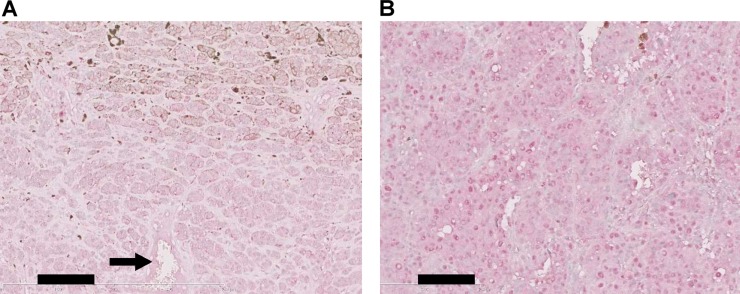
Immunohistochemical analysis of TRPM5 expression in clinical specimens of human melanoma Representative results of immunohistochemical analysis of TRPM5 expression in primary melanoma (skin) (**A**) with immunoreactivity evaluated as +++ and secondary melanoma (lung) (**B**) with immunoreactivity evaluated as +++. The arrow indicates endothelial cells, defined as the standard for immunoreactivity. Data are summarized in Table 1. Scale bar, 100 µm.

**Figure 9:**
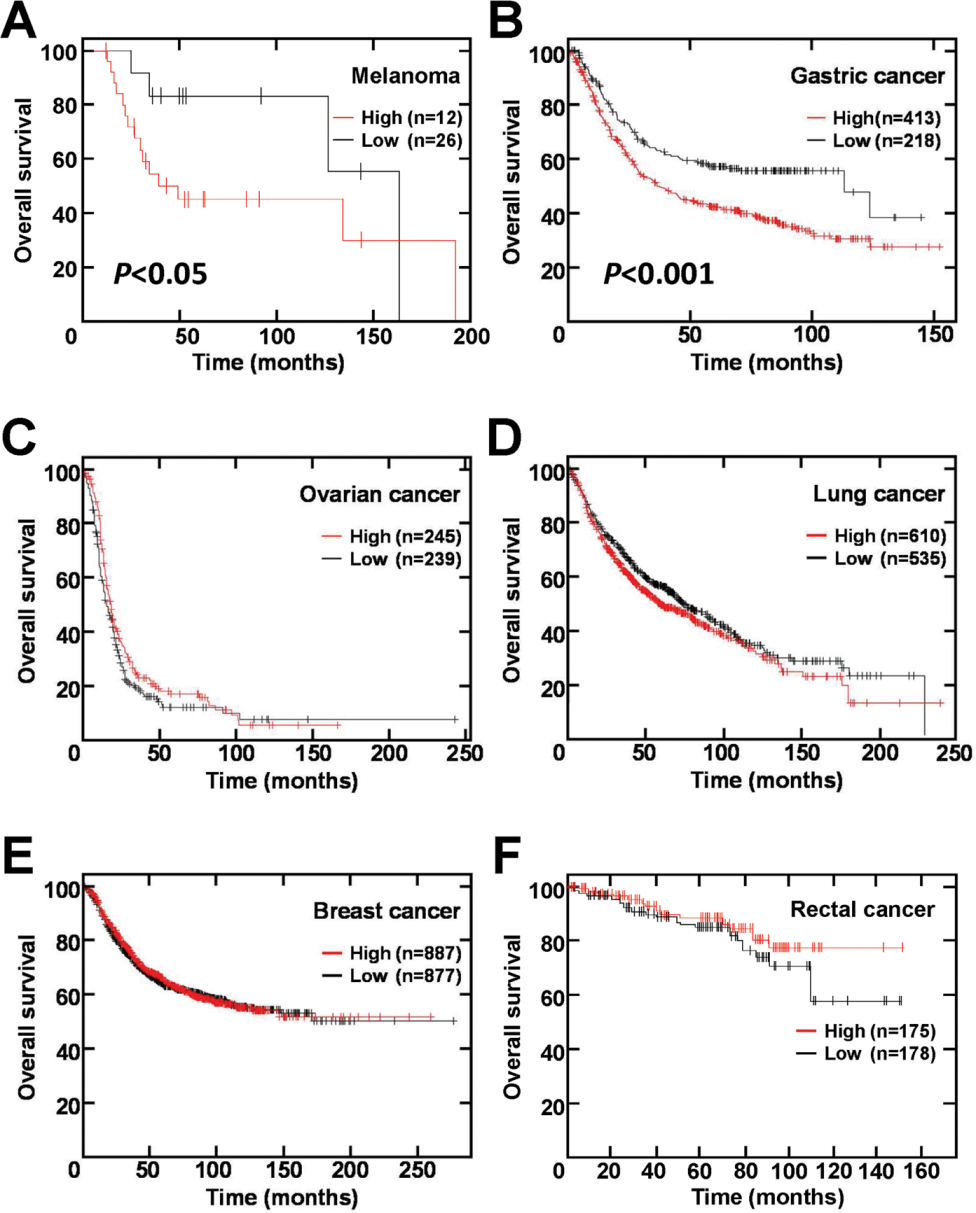
*TRPM5* mRNA expression correlates with survival rate of patients with melanoma and gastric cancer (**A**) Melanoma. (**B**) Gastric cancer. (**C**) Ovarian cancer. (**D**) Lung cancer. (**E**) Breast cancer. (**F**) Rectal cancer. Data were obtained from the Gene Expression Omnibus (GEO; https://www.ncbi.nlm.nih.gov/geo) for melanoma (GSE19234) and rectal cancer (GSE87211) and from the KM-plotter database (http://kmplot.com/analysis/index.php?p=background) for gastric, ovarian, breast, and lung cancer.

We further checked whether TRPM5 expression predicts clinical outcome for human melanoma patients. Immunohistochemical staining of clinical specimens showed that all primary (Figure 8A) and secondary (Figure 8B) lesions were positive for TRPM5 protein, with no significant difference in TRPM5 protein levels between primary and metastatic sites (Table 1). *In silico* analysis of TRPM5 mRNA levels in clinical samples from cancer patients were correlated with overall survival using an open database. High TRPM5 expression in melanoma patients was associated with shorter survival time (Figure 9A). Interestingly, this correlation was much clearer in gastric cancer patients (Figure 9B), but was not observed in patients with ovarian (Figure 9C), lung (Figure 9D), breast (Figure 9E) and rectal (Figure 9F) cancer. These findings suggested that TRPM5 expression is closely associated with prognosis in patients with melanoma and gastric cancer, but not in patients with other tumor types.

**Table 1:** Immunocytochemistry of TRPM5 expression in melanoma patients

Site	Intensity	
	++	+++
Primary^a)^	6 (40%)	9 (60%)
Secondary^b)^	9 (47%)	10 (53%)

^a)^13 in skin, 1 in eyeball, and 1 in nasal cavity

^b)^8 in lymph node, 6 in skin, 2 in brain, 1 in soft tissue, 1 in lung, and 1 in liver.

*P* = 0.468

## DISCUSSION

We previously reported that acidic pH_*e*_ induces MMP9 production in mouse B16 melanoma cells and cell invasion through type IV collagen sheets, as well as activating intracellular signaling pathways [4]. These pathways involve Ca^2+^-triggered PLD activation followed by activation of MAP kinases (extracellular signal-regulated kinase1/2 and p38) and NF-κB [4]. Acidic sphingomyelinase is also involved in acidic pH_*e*_ induction of NF-κB [5]. PLD1, but not PLD2, was shown to contribute to acidic pH_*e*_-induced MMP-9 signaling, with RhoA activating PLD1 [6]. The present study further explored this acidic pH_*e*_ sensing system, finding that TRPM5 was involved in acidic pH_*e*_-induced MMP-9 expression. The TRPM5 inhibitor TPPO significantly inhibited spontaneous metastasis to the lungs. In addition, *in silico* analysis showed a significant correlation between high levels of TRPM5 expression and shorter overall survival in patients with melanoma and gastric cancer, but not in patients with cancers of the lung, breast, and rectum. These findings suggest that TRPM5 may be a potential therapeutic target, as its inhibition may prevent metastasis and prolong overall survival of patients with melanoma and gastric cancer. This study also found that *Trpm5* mRNA expression was induced by acidic pH_*e*_ but that this induction was not inhibited by TPPO treatment.

In mice, most *Trpm5* mRNA is present in organs such as the taste buds, stomach, and small intestine [29]. The present study showed that overall survival of gastric cancer patients was significantly shorter in patients with high than low levels of TRPM5. Chronic gastritis and peptic ulcers are closely associated with the development of gastric cancer [30], as tumor cells seem to selectively grow in these environments. Taken together, these findings indicate that TRPM5 may be associated with cell adaptation to acidic pH_*e*_.

Molecular targeting chemotherapy agents inhibit the growth of tumor cells and/or induce their apoptosis. These agents include imatinib and ponatinib, which inhibit BCR-ABL tyrosine-kinase and are used to treat chronic myelogenous leukemia (CML) and gastrointestinal stromal tumors (GISTs) [31, 32]; gefitinib, erlotinib, and afatinib, which inhibit epidermal growth factor receptor (EGFR) tyrosine kinase and are used to treat lung cancer [33]; and trastuzumab, an anti-HER2 antibody, which is used to treat breast cancer [34]. Inhibitory agents targeting the Warburg effect include echinomycin (NSC-13502), which targets HIF1 [35]; HOE 642 (Caripride) [23, 36], zoniporide (CP-597,396) [37], EMD 84021, EMD 94309, and EMD 96785 (eniporide) [38], which target Na^+^/H^+^-exchanger 1 (NHE1); and sulfonamide (sulfamates), coumarins, and girentuximab (monoclonal antibody against G250 antigen), which target CAIX [39]. These drugs inhibit the generation of acidic pH_*e*_ microenvironments. Because acidic pH_*e*_ induces MMP-9 expression and cell migration into type IV collagen sheets [2], we hypothesized that acidic pH_*e*_ is not only a consequence of the Warburg effect but a trigger of the metastatic phenotype. Our hypothesis has been confirmed by evidence, from not only our group but also other groups, showing that acidic pH_*e*_ induces the disruption of extracellular matrices, as well as tumor invasion and metastasis [40–48]. Agents that target the acidic pH_*e*_ sensing machinery, such as ASIC1a [49, 50] and TRPV1 (vanilloid receptor) [51], can prevent tumor growth. TRPV1, which can be activated by chemical stimuli such as capsaicin, H^+^, and heat, is a pain receptor [52–54]. Administration of capsazepine, an antagonist for TRPV1, has been found to prevent cutaneous vasodilation [55], suggesting that capsazepine may be a promising candidate for prevention of tumor growth, although its ability to prevent metastasis remains unclear. On the other hand, we showed that constitutive expression of TRPM5 is apparently uninvolved in tumor growth *in vivo*. Moreover, TPPO treatment did not reduce tumor growth *in vivo*; rather, high concentrations of TPPO slightly enhanced tumor growth *in vitro,* perhaps due to a side effect of TPPO. Clinically, therefore, TRPM5 inhibitors may be administered together with cytostatic and/or cytotoxic agents. Because TRPM7 contributes to the invasiveness of glioblastoma *in vitro* [56], antagonism of TRPM7 may prevent tumor metastasis.

TRPs have been divided into seven sub-families: TRPC (canonical), TRPV (vanilloid), TRPM (melastatin), TRPML (mucolipin), TRPP (polycystin), and TRPA (ankyrin transmembrane protein) and TRPN (nomPClike) [15]. *In silico* analysis of the survival rates of 14 types of tumor expressing 27 types of TRPs showed that high levels of expression of TRPC4, TRPM3, TRPP1, and TRPA1 correlated with significantly better survival rate in patients with clear cell renal cell carcinoma while TRPC4 was found to be closely associated with incidence of head and neck cancer [57]. That study, however, did not analyze TRPM5 expression in melanoma and gastric cancer. The present study found that high levels of TRPM5 expression correlated with significantly shorter overall survival. Unexpectedly, however, immunohistochemical staining for TRPM5 showed no correlation with metastasis. This may be explained by differences between mRNA and the protein half-life of TRPM5. The single nucleotide polymorphism (SNP) database of the National Center for Biotechnology Information of the U.S. National Library of Medicine (http://www.ncbi.nlm.nih.gov/snp/?term=TRPM5) currently includes 10344 SNPs for human TRPM5, suggesting that mutations in *TRPM5* may contribute to tumor metastasis. Further studies are needed to clarify the relationship between the polymorphism and the channel activity of TRPM5. It is also necessary to determine which monovalent cation ion permeabilizing through TRPM5 is involved in acidic pH_*e*_ signaling for MMP-9 production.

In conclusion, this study is the first to show that TRPM5 is associated with acidic pH_*e*_-signaling in *Mmp9* mRNA expression in B16-BL6 melanoma cells and that pharmacological inhibition of TRPM5 successfully inhibited their spontaneous metastasis. TRPM5 may be a promising target to prevent lung metastasis of some kinds of tumors.

## MATERIALS AND METHODS

### Reagents

Dulbecco’s modified Eagle’s medium (DMEM), Ham’s F12 medium, and High Capacity RNA-to-cDNA kits were purchased from Life Technologies (Grand Island, NY, USA). Rhodamine-phalloidin was from Cytoskeleton, Inc. (Denver, CO, USA). The Dual-luciferase Assay System was from Promega (Madison, WI, USA). TPPO and glucose CII test kit were from Wako (Tokyo, Japan). Isogen total RNA extraction kits were purchased from Nippon Gene (Tokyo, Japan). Ex-*Taq* polymerase, SYBR Premix Ex *Taq* II and Xfect™ transfection reagent were from Takara Bio (Tokyo, Japan). Rhodamine-phalloidin was from Cytoskeleton, Inc. (Denver, CO, USA). Fetal bovine serum (FBS) was from Hyclone (South Logan, UT, USA). The blocking reagent N102 was from NOF (Tokyo, Japan). Immobilon-P PVDF membranes and chemiluminescence reagent were from Merck Millipore (Billerica, MA, USA). Anti-TRPM5 polyclonal antibody was from Alomone Labs (Jerusalem, Israel), and anti-tubulin monoclonal antibody was from Santa Cruz (Dallas, TX, USA). Avidin-conjugated horseradish peroxidase (HRP) was from Bio-Rad (Hercules, CA, USA). Histofine immunohistochemical staining kits were from Nichirei Biosciences (Tokyo, Japan).

### Vectors and vector construction

The plasmid pMEi8FL3, into which the mouse *Trpm5* gene had been inserted, was the kind gift of Dr. Keiko Abe (University of Tokyo, Tokyo, Japan) [58]. The pIRESneo3 vector was purchased from Clontech (Takara Bio). The *Trpm5* gene from pMEi8FL3 was subcloned into the *Eco*RI/*Not*I site of pIRESneo3.

The NF-κB-driven luciferase reporter construct (pNFκB-Luc) was purchased from Panomics (Fermont, CA, USA). The cytomegalovirus-driven *Renilla* luciferase reporter vector (pGL4.75[hRluc/CMV]) for normalization of transfection efficiency was from Promega.

### Cells and cell culture

Mouse B16-F1, B16-F10, and B16-BL6 melanoma cells [2–6] were cultured in basal medium, consisting of a 1:1 mixture of DMEM and F12 supplemented with 15 mM HEPES, 4 mM H_3_PO_4_ 1.8 g/l NaHCO_3_, 100 units/mL penicillin G, and 0.1 mg/ml streptomycin sulfate, adjusted to pH 7.4 with NaOH or pH 5.9 with HCl [5, 6]. For serial culture, cells were washed twice with Ca^2+^-Mg^2+^-free Dulbecco’s phosphate-buffered saline [PBS(-)], passaged with trypsin/EDTA and cultured in basal medium at pH 7.4 supplemented with 10% fetal bovine serum (FBS) at 37°C in a humidified atmosphere in a 5% CO_2_ incubator.

### NF-κB activity

NF-κB activity was determined by transfection of an NF-κB-driven luciferase reporter vector (pNFκB-Luc) along with a CMV-driven *Renilla* luciferase reporter vector (pGL4.75[hRluc/CMV]) as described, with some modifications [4]. Briefly, B16-BL6 cells in 12-well plates were transfected with 2.5 μg of a 30:1 mixture of pNFκB-Luc and pGL4.75[hRluc/CMV] per well using Xfect™. Reporter activity was measured using the dual-luciferase reporter assay system according to the manufacturer’s protocol.

### *Trpm5* mRNA knock down

An siRNA oligonucleotide, 5′-GUUGAUGAGGCUCGUGUGAAU-3′ (Nippon Gene, Tokyo, Japan) was constructed to knock down *Trpm5* mRNA expression in B16-BL6 cells.

### RT-PCR and RT-qPCR

Total RNA was purified with Isogen and reverse-transcribed to cDNA using a High-Capacity cDNA Reverse Transcription Kit. The target sequence was amplified by Ex-*Taq* polymerase and SYBR Premix Ex *Taq* II in a Thermal Cycler Dice Real Time System (TP-870, Takara Bio) using specific primer sequences for *Mmp9* (85 bp), 5′-gcc ctg gaa ctc aca cga ca-3′ (upstream) and 5′-ttg gaa act cac acg cca gaa-3′ (downstream); *Mmp2* (125 bp), 5′-aac ggt cgg gaa tac agc ag-3′ (upstream) and 5′-gta aac aag gct tca tgg ggg-3′ (downstream); *Vim* (vimentin, 198 bp), 5′-gga cgt ttc caa gcc tga cct c-3′ (upstream) and 5′-ccg gta ctc gtt tga ctc ctg c-3′ (downstream); *Cdh2* (N-cadherin, 132 bp) 5'-gca ttc agc acc cac ctc agt c-3' (upstream) and 5'-tca gca tgg tac ctg cgt gga g-3' (downstream); and *Actb* (b-actin, 85 bp), 5′-cat ccg taa aga cct cta tgc caa c-3′ (upstream) and 5′-atg gag cca ccg atc cac a-3′ (downstream) [7]. The level of expression of each target gene was normalized relative to the level of *Actb* mRNA in the same samples.

### Zymography

MMP-9 activity was determined by zymography as described [2, 3, 7]. Briefly, cells were cultured in a serum-free medium for 24 h. The proteins in the conditioned medium (CM) were precipitated with acetone and separated by electrophoresis in gelatin-containing 7.5% polyacrylamide gels in the presence of sodium dodecyl sulfate (SDS), without prior heating or reduction. After electrophoresis, the gels were treated with 2.5% Triton-X100 in Tris-HCl (pH 7.5) / 5 mM NaCl to remove SDS, incubated in 50 mM Tris-HCl (pH 7.5) / 10 mM CaCl_2_ for 24 h, and stained with Coomassie Brilliant Blue R-250.

### Western blot analysis

Cells were lysed in RIPA buffer containing inhibitor cocktail as described [5, 59]. Proteins in cell lysate were separated by SDS-acrylamide electrophoresis and transferred onto Immobilon-P polyvinylidene fluoride membranes. The resultant membranes were blocked with TBS-T (20 mM Tris-HCl [pH 7.5], 150 mM NaCl, and 0.05% Tween 20) containing 20% blocking regent N102 and treated sequentially with primary antibody, biotin-conjugated secondary antibody, and avidin-conjugated horseradish peroxidase. Signals were detected with enhanced chemiluminescence reagents. Anti-a-tubulin antibody was used as a loading control.

### Actin stress fiber staining

Cells were seeded on glass coverslips in DMEM/F12 (pH 7.4) supplemented with 10% FBS and incubated overnight. The cells were washed twice with PBS(-) and once with serum-free DMEM/F12 (pH 7.4), incubated with DMEM/F12 (pH 7.4 or 5.9) for 24 h, fixed in 4% paraformaldehyde/PBS(-) and permeabilized by exposure to 0.2% Triton X-100/PBS for 2 min [7]. After washing with PBS(-), the cells were incubated with 20% N102 blocking reagent in PBS(-) and with rhodamine-phalloidin (1 U/ml) for 30 min at room temperature. Fluorescent signals were detected using a fluorescence microscope (Axio Imager Z1; Carl Zeiss, Oberkochen, Germany).

### CBG level

CBG level was measured using a glucose CII test kit according to the manufacturer’s instructions.

### Tumor size measurement

Tumor volumes were calculated using the formula: 1/6 × π × Length (mm) × Width (mm) × Height (mm) [60].

### Lung metastasis

All experimental protocols on animals were performed in accordance with the guidelines of the Ministry of Education, Culture, Sports, Science and Technology and the Ministry of Health, Labor and Welfare of Japan and were approved by the Animal Experimental Committee of Ohu University (Koriyama, Japan). Throughout all experiments, mice were anesthetized with a mixture of medetomidine-hydrochloride, midazolam, and butorphanol-tartrate [61, 62].

B16-BL6 cells were trypsinized, resuspended in serum-containing DMEM/F12 (pH 7.4), and incubated at 37°C for 1 h. The cells were washed twice with PBS(-), and resuspended in ice cold PBS(-). In experimental metastasis assays [63], 5 × 10^6^ cells/200 µl/mouse were injected into the tail vein of 7-week-old male C57BL/6 mice (Clea Japan, Tokyo, Japan). Three weeks later, the mice were sacrificed, their lungs were removed and the numbers of metastatic foci counted. Data are representative of two independent experiments.

In spontaneous metastasis assays [64–69], 5 × 10^5^ cells/30 µl/mouse were injected into the left footpad of anesthetized 7-week-old male C57BL/6 mice. Beginning 2 days later, TPPO (10 mg/kg), dissolved in DMSO [27, 70], was subcutaneously administered every other day. After 3 weeks, the mice were anesthetized and their left feet, containing the primary tumors, were amputated. After an additional 4 weeks, the mice were sacrificed, their lungs were removed and the numbers of metastatic foci counted. Data are combined from three independent experiments.

### Immunohistochemical staining of TRPM5 in pathological specimens

Biopsy or surgically resected specimens were collected from 30 patients diagnosed with melanoma from 2002 to 2012 at Fukuoka University Hospital, Fukuoka, Japan. None had received chemotherapy, radiotherapy, or any other adjunct to surgery. Fifteen patients had primary tumors, including 13 in the skin, one in the eyeball, and one in the nasal cavity; and 15 had metastases, including eight in lymph nodes, six in skin, two in the brain, and one each in the lung and liver. Paraffin-embedded samples (5 μm thick) were pre-treated by incubation in a microwave oven for 10 min in 10 mM citrate buffer (pH 6.0). The samples were immunohistochemically stained with anti-Trpm5 polyclonal antibody (1:100) using a Histofine immunohistochemical staining kit (Nichirei, Tokyo, Japan). Color was developed with alkaline phosphatase and Fast Red. Specimens were counter stained for nuclei with methyl green. The intensity of staining was compared with that in endothelial cells (ECs) and scored as stronger than ECs (+++), equal to ECs (++), weaker than ECs (+), and negative (–).

The clinical study confirmed to Japanese ethical guidelines for clinical and epidemiological studies, based on the Declaration of Helsinki, and was approved (reference #16–4-10) by the Ethics Committee of Fukuoka University (Fukuoka, Japan). All patients provided written informed consent.

### *In silico* analysis for association of patient outcome with gene expression

Gene expression data were downloaded from open databases, including the Gene Expression Omnibus (GEO; https://www.ncbi.nlm.nih.gov/geo) databases GSE19234 for melanoma and GSE87211 for rectal cancer; and the KM-plotter database (http://kmplot.com/analysis/index.php?p=background) for gastric, ovarian, breast, and lung cancer [71].

### Statistical analysis

Results were expressed as mean ± SE. Student’s *t*-test was used to compare two independent samples. Data of *in vitro* assays were representative of two or more independent experiments, each of which contained triplicate samples, unless otherwise noted. Immunohistochemistry results were compared by Fisher’s exact test, and overall survival was compared by the Wilcoxon test. A *P* value < 0.05 was considered statistically significant.

## SUPPLEMENTARY MATERIALS FIGURES


