# Supplementary material for: TRPM5 mediates acidic extracellular pH signaling and TRPM5 inhibition reduces spontaneous metastasis in mouse B16-BL6 melanoma cells
Source: Oncotarget. 2017 Sep 11;8(45):78312–26. doi: 10.18632/oncotarget.20826 (PMC5667964; doi:10.18632/oncotarget.20826)
Supplement: Supplementary file 1 [file oncotarget-08-78312-s001.pdf]

TRPM5 mediates acidic extracellular pH signaling and TRPM5 inhibition reduces spontaneous metastasis in mouse B16-BL6 melanoma cells

SUPPLEMENTARY MATERIALS

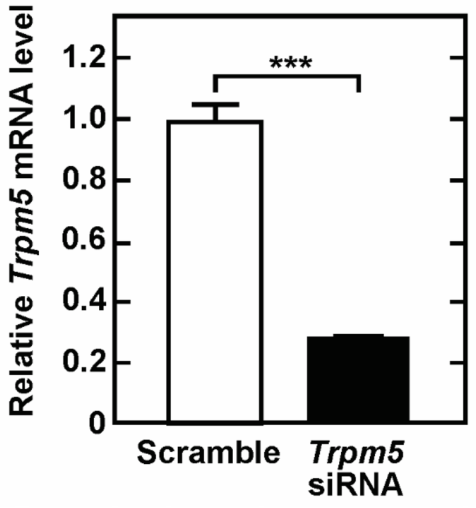

Supplementary Figure 1: Reduction of *Trpm5* mRNA by transfection of *Trpm5* siRNA. After transfection of *Trpm5* siRNA, total RNA was extracted, reverse-transcribed, and quantified by qPCR. Data were calculated relative to scramble control and represented as mean ± SE in triplicate assay. ***$P < 0.001$.

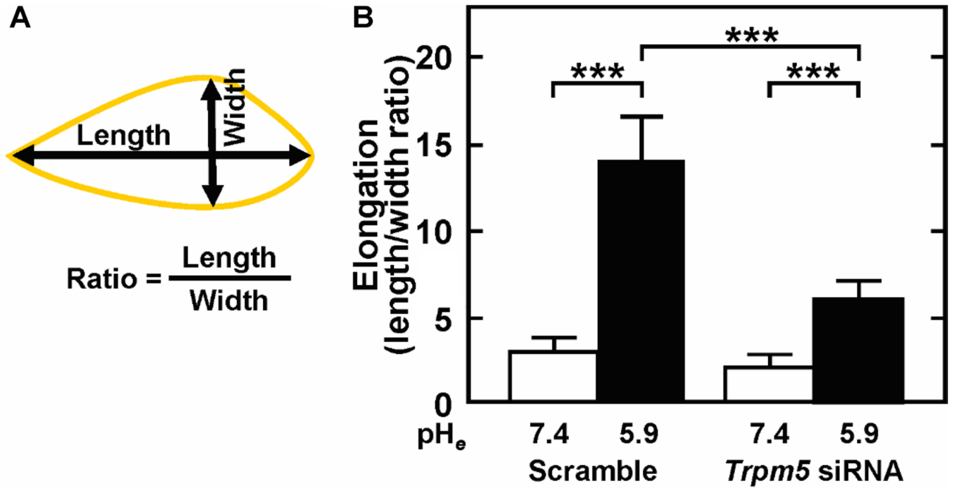

Supplementary Figure 2: Introduction of *Trpm5* siRNA reduces acidic pH$_e$-altered morphology to fibroblastic. Inhibition of acidic pH$_e$-induced elongation by transfection of *Trpm5* siRNA. Following transfection, the cells were incubated in serum-free medium at neutral and acidic pH. Cell elongation was determined as length/width ratio (**A**) and represented as mean ± SE in quadruplicate assay of more than 50 cells (**B**). ***$P < 0.001$.

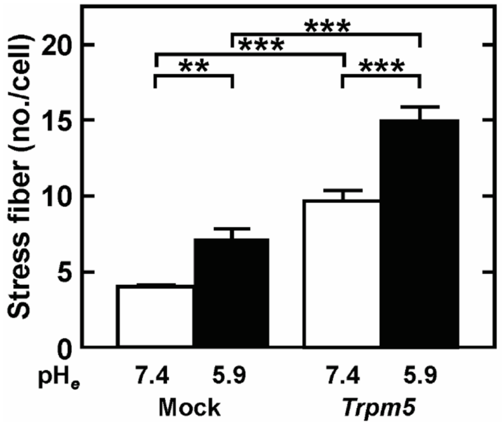

Supplementary Figure 3: Constitutive expression of *Trpm5* mRNA increases the induction of actin reorganization by acidic pH$_e$. After rhodamine-phalloidin staining, numbers of well-organized stress fibers in *Trpm5* (#16) and Mock (#9) transfectants were counted and expressed as the counts per cell in quintuplicate assay of more than 35 cells. ***P* < 0.01, ****P* < 0.001.

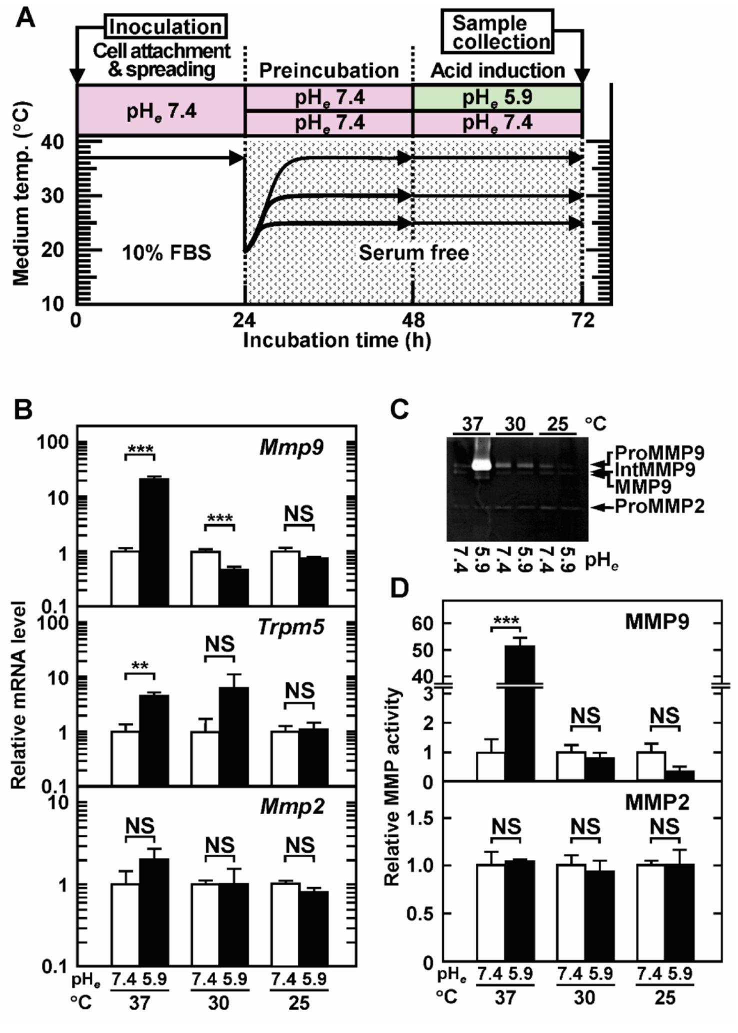

Supplementary Figure 4: Thermal inactivation of channel activity of TRPM5 reduced acidic pH$_e$ induction of MMP-9 production. (**A**) Time line illustrating course of treatment of BL6 cells: pH$_e$ and temperature of media. (**B**) RT-qPCR for *Mmp9, Mmp2* and *Trpm5* mRNA. (**C**) Zymographic analysis for MMP-9 and -2. (**D**) Densitometric analysis of zymography for MMP-9 and -2 in triplicate assay including (C) as the representative. All data were expressed as relative to pH$_e$ 7.4 at each temperature. ***P* < 0.01, ****P* < 0.001. NS, not significant.
